# How Does the Environment Affect Wheat Yield and Protein Content Response to Drought? A Meta-Analysis

**DOI:** 10.3389/fpls.2022.896985

**Published:** 2022-06-30

**Authors:** Chenxi Wan, Pengfei Dang, Licheng Gao, Jiale Wang, Jincai Tao, Xiaoliang Qin, Baili Feng, Jinfeng Gao

**Affiliations:** State Key Laboratory of Crop Stress Biology for Arid Areas, College of Agronomy, Northwest A&F University, Xianyang, China

**Keywords:** drought stress, protein, wheat, yield, drought response

## Abstract

Wheat (*Triticum aestivum* L.) is one of the most significant cereal crops grown in the semi-arid and temperate regions of the world, but few studies comprehensively explore how the environment affects wheat yield and protein content response to drought by means of meta-analysis. Therefore, we collected data about grain yield (GY), grain protein yield (GPY), grain protein content (GPC), and grain nitrogen content (GNC), and conducted a meta-analysis on 48 previously published data sets that originate from 15 countries. Our results showed that drought significantly decreased GY and GPY by 57.32 and 46.04%, but significantly increased GPC and GNC by 9.38 and 9.27%, respectively. The responses of wheat GY and GNC to drought were mainly related to the drought type, while the GPY was mainly related to the precipitation. The yield reduction due to continuous drought stress (CD, 83.60%) was significantly greater than that of terminal drought stress (TD, 26.43%). The relationship between the precipitation and GPY increased in accordance with linear functions, and this negative drought effect was completely eliminated when the precipitation was more than 513 mm. Sandy soils and high nitrogen application level significantly mitigated the negative effects of drought, but was not the main factor affecting the drought response of wheat. Compared with spring wheat, the drought resistance effect of winter wheat was more obvious. Evaluation of these models can improve our quantitative understanding of drought on wheat yield and food security, minimizing the negative impact of drought on crop production.

## Introduction

Drought has been the major pressure on crop production due to the reduced precipitation and rising temperature, threatening global food security (Fahad et al., 2021b). Recently, nearly half of the crop production areas are frequently affected by either terminal drought stress (TD) or continuous drought stress (CD) worldwide, representing drought after flowering and drought throughout the growth period, respectively, and resulting in sharp declines in cereal yields (Batool et al., 2019). Wheat (*Triticum aestivum* L.) is considered to be the most widely grown cereal in the world and the main source of protein (Abdel-Aal and Hucl, 2002; Giraldo et al., 2019). Drought not only reduces the grain yield (GY) but also changes the grain protein content (GPC) (Farooq et al., 2014; Magallanes-López et al., 2017).

The negative effects of drought on wheat growth and development depended on the external environment (Fahad et al., 2021a). Climatic factors (temperature, precipitation, and drought type), soil factors (soil type, soil organic matter content), and nitrogen fertilizer input all affected wheat yield and quality. Several studies have shown that the extreme depletion of soil moisture and plant carbohydrate reserves under drought conditions leads to the major challenge for the yield and quality of any crop is water supply throughout the growing period (Angus and Herwaarden, 2001; Selim et al., 2019). On the one hand, insufficient water supply results in the reduction of carbohydrate synthesis of crops, further resulting in lower grain yield and protein yield. In addition, carbohydrate content is inversely proportional to protein content (Sehgal et al., 2018), so a decrease in carbohydrate content under drought conditions leads to an increase in grain protein content. On the other hand, do Nascimento Silva et al. (2020) and Fahad et al. (2021d) believed that nitrogen fertilizer input also mitigates the negative effects of drought on wheat production and enhance plant tolerance to a certain extent.

Meta-analysis is a method of quantitatively comparing the results of numerous studies, which summarizes the range of projected results and evaluates the consensus (Hedges and Curtis, 1999). However, most meta-analyses on wheat response to drought had focused on yield and agronomic traits (Nawaz et al., 2015; Abdel-Motagally and El-Zohri, 2018; Zhang et al., 2018). External environmental factors such as climatic factors, soil factors, and nitrogen inputs can affect (aggravate or mitigate) the response of wheat to drought. However, the extent of wheat yield and protein content reduced by drought and the main factors affecting drought response have not been fully studied.

Therefore, we collected data about GY, GPC, GNC, and GPY from 48 articles published before February 2020 explored the main environmental factor affecting the response of wheat to drought, and considered how this factor affects drought response. In this study, we utilized a comprehensive meta-analysis on the following aspects: (1) estimated the effects of different drought types (terminal drought stress (TD) or continuous drought stress (CD)); various N application levels (low: 0–100 kg/ha, medium: 100–200 kg/ha, and high: >200 kg/ha); different soil types (sandy, loam, clay); different wheat types (winter wheat, spring wheat); mean annual precipitation and mean annual temperature on the response of GY, GPY, GPC, GNC of wheat under drought stress; (2) determined the main external factors of the response of GY, GPY, GPC, GNC under drought stress; and (3) analyzed how this factor affects drought response. We hypothesized that the responses of wheat to different drought types could be different; nitrogen fertilizer had a certain mitigation effect on the drought response of wheat but may not be the decisive factor. Evaluation of these models can improve our quantitative understanding of drought on wheat yield and food security, minimizing the negative impact of drought on crop production.

## Materials and Methods

### Database Construction

A database was built of drought stress on GY, GPY, GPC, GNC of wheat by surveying peer-reviewed literature published before 2020 (February) within the Web of Google Scholar (http://scholar.google.com.au) and Science (ISI, USA, http://apps.webofknowledge.com/). The keywords of the search were drought stress, water deficit, water stress, and wheat protein, to identify studies that covered the targeted research content. The articles were selected based on the following criteria: (1) studies must include control and experimental treatments, (2) data must include at least one of the relevant parameters in GY, GPY, GPC, GNC, (3) data must cover sample size (N), means (M) and a variance measurement [standard deviation (SD), standard error (SE), or coefficient of variation (CV)] for all control and treatment groups, (4) data were included only when N application (chemical fertilizer input) was clearly indicated and different from zero. If provided, data about GY and GPC were collected; GPY, if not clearly reported, was calculated by multiplying GY by GPC, (5) regarding the Fp, if not given in the article, was calculated as 5.7, (6) when two types of drought stress (TD or CD) were applied in a study, the data representing a severe drought treatment were selected, respectively. The means, SD or SE, and the number of observations were collected from the text, tables, and figures from each article, (7) in the case where the temperature, precipitation, means, and variance can only be obtained from the figures, the data were digitized and gathered using the Getdata Graph Digitizer software (http://getdata-graph-digitizer.com/).

### Composition of the Database

We collected data about grain yield (GY), grain protein yield (GPY), grain protein content (GPC), and grain nitrogen content (GNC) and conducted a meta-analysis on 48 previously published data sets that originate from 15 countries: 31 publications from Asia, 4 from Europe, 7 from North America, 3 from Africa, 2 from Australia, and 1 from South America. The abbreviations involved in the studies are divided into the following parts (Table 1).

**Table 1:** The abbreviation of classification of drought and the indicators of yield, protein yield, and protein content as reported in this meta-analysis.

**Parameter Abbreviation**	**Description**
TD	Terminal drought stress
CD	Continuous drought stress
N	Nitrogen
GY	Grain yield
GPY	Grain protein yield
GPC	Grain protein content
GNC	Grain nitrogen content
Fp	Nitrogen-to-protein conversion factor

The data extracted were presented in Supplementary Table S1. These also included information such as mean annual temperature, mean annual precipitation, soil organic matter content, and N application level. Data only expressed in graphs were extracted by GetData Graphic Digitizer 2.25.0.20 (http://www.getdata-graph-digitizer.com/).

### Statistical Analyses

To characterize the response of wheat yield, protein content, and nitrogen content of grains to drought, we quantified the effect of wheat on drought stress by using a meta-analysis. The natural logarithm (ln) of the response ratio (R) is a formal quantitative statistical method to quantify the effect of wheat on drought stress (Hedges and Curtis, 1999). For the variables involved in the article, R represented the ratio of the value in the drought stress treatment (X^E^) to the adequate irrigation control (X^C^), and the following equationwas used to calculate the effect size:


(1) $$\begin{array}{r} \ln\;R=\ln\;\left(\frac{X^{E}}{X^{C}}\right)=\ln\;\left(X^{E}\right)-\ln\;\left(X^{C}\right) \end{array}$$

The variation (v) of ln R was calculated by the following equation:


(2) $$\begin{array}{r} v\ln\;R=\frac{{\left(S^{E}\right)}^{2}}{N^{E}{\left(X^{E}\right)}^{2}}+\frac{{\left(S^{C}\right)}^{2}}{N^{C}{\left(X^{C}\right)}^{2}} \end{array}$$

where S^E^ and S^C^ were the standard deviation of treatment and control, respectively, and N was the sample size (Rosenberg et al., 1997). To better explain the results, the lnR was converted to a percentage, and calculated by the following equation:


(3) $$\begin{array}{r} E=\left[\text{exp}\left(\ln\;R\right)-1\right]\times 100 \end{array}$$

The corresponding value would be converted to SD by the equation, if that is performed as SE or coefficient of variation (CV):


(4) $$\begin{array}{r} SD=SE\times \sqrt{N} \end{array}$$

(5) $$\begin{array}{r} SD=CV\left(\%\right)\times \bar{X} \end{array}$$

where $\bar{\text{X}}$ was the mean value of treatment. The average CV was calculated in each data set using $\bar{\text{X}}$ and the SE was obtained by (4) when SD or SE was not reported.

The statistical software MetaWin 2.1 was used to perform 9,999 iterations of resampling in the meta-analysis. If the 95% CI of the variable did not overlap zero, the variable was considered to increase significantly (average response rate > 0). Otherwise, drought treatment had no significant effect on this variable (average response rate <0) (*P* < 0.05) (Wang et al., 2013; Schwarzer et al., 2015).

First, we estimated the effects of drought types (TD and CD), N application levels (low: 0–100 kg/ha, medium: 100–200 kg/ha, and high: >200 kg/ha), soil types (sandy, loam, clay), wheat type (winter wheat, spring wheat), mean annual precipitation, and mean annual temperature on the response of GY, GPY, GPC, GNC of wheat under drought stress. Then, the total heterogeneity (Q_T_) and overall response ratio were calculated to test whether the variances were significantly different or not. The data were heterogeneous and further analyzed through single-factor categorical analyses if *P* < 0.05. The between-group heterogeneity (Q_B_) was calculated for various types of variables according to the classification (Curtis and Wang, 1998; Chandrasekaran et al., 2014).

Second, we explored the main controlling factors of wheat GY, GPY, GPC, and GNC responses to drought through a random forests (RF) model. The RF model provided a single prediction that had low bias and variance by generating a large number of trees (Grömping, 2009). Among them, MSE stands for mean squared error. Each predictor variable was randomly assigned, and the more important the variable was, the greater error of the model prediction after its value was randomly replaced. Finally, we explored the main environmental factor affecting the response of wheat to drought, and considered how this factor affects drought response. All response functions were compared and screened based on R^2^, and log (natural logarithm) was used to transform the response ratio.

## Results

### Variations in GY and GPY Response to Drought

**Figure 1:**
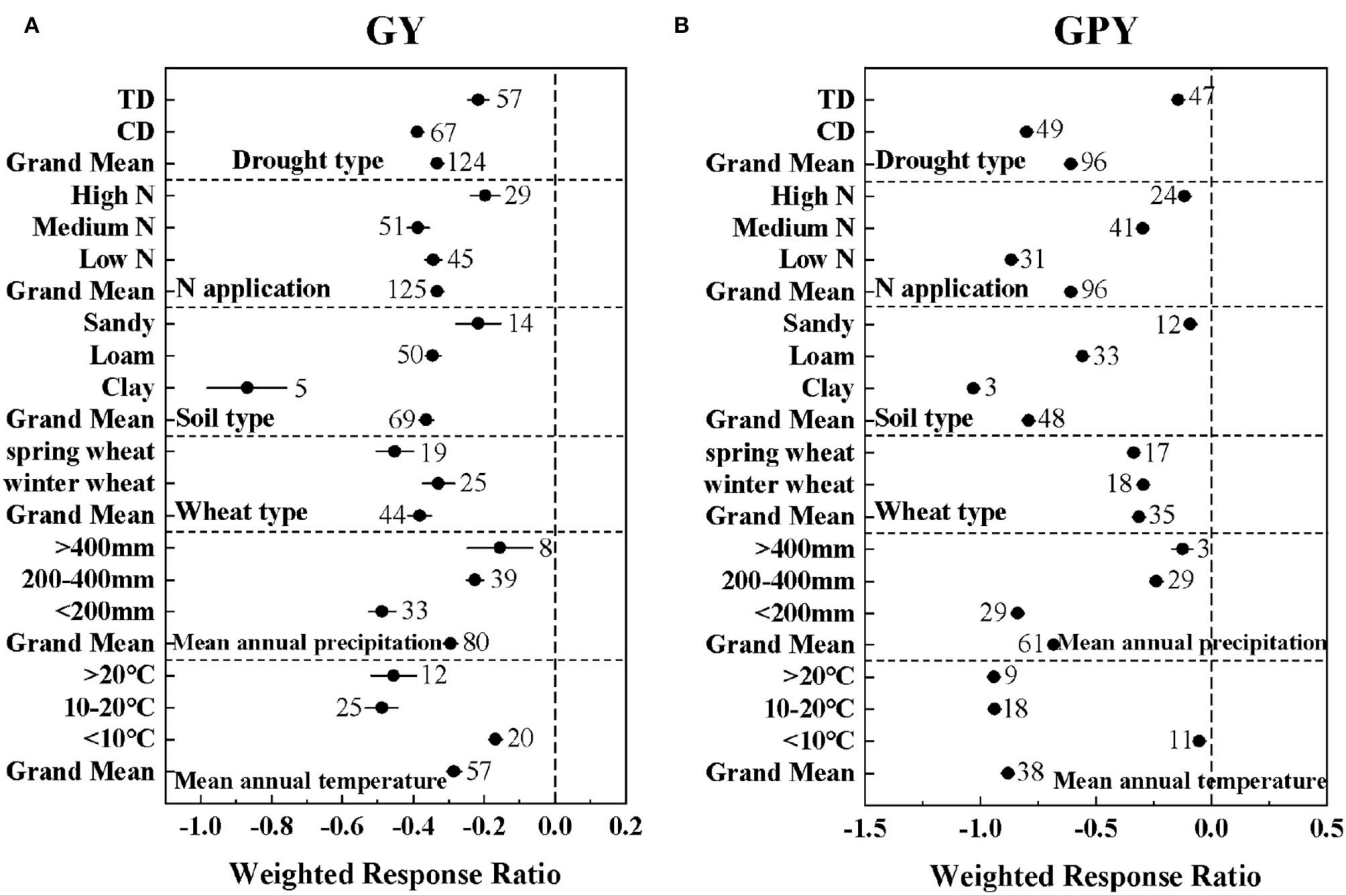
The effect of drought type (TD: Terminal drought stress; CD: Continuous drought stress), N application level [Low N (0–100 kg/ha); Medium N (100–200 kg/ha); High N (>200 kg/ha)], soil type, wheat type, mean annual precipitation, and mean annual temperature on the lnRRs of **(A)** GY: grain yield and **(B)** GPY: grain protein yield. The sample size of each variable is noted beside each bar. The effect of drought is significant if the ±95% confidence intervals of effect size do not overlap zero.

Drought significantly reduced GY by 57.32% and GPY by 46.04% (Supplementary Table S3). Meanwhile, the meta-regression analysis showed that GY and GPY were affected by many factors under drought conditions, including drought type, N application level, soil type, wheat type, mean annual precipitation, and mean annual temperature (Figure 1). The alleviation effect of low and medium nitrogen levels on wheat GY and GPY was not significant. High nitrogen levels and sandy soils significantly mitigated the negative effects of drought on GY and GPY. Both CD and TD significantly reduced GY by 83.60 and 26.43%, respectively (Supplementary Table S3). The climatic conditions with mean annual precipitation of > 400 mm and mean annual temperature of <10°C significantly increased GY and GPY. Meanwhile, the negative response values of winter wheat to GY and GPY (effect size = −0.33 ± 0.046; −0.29 ± 0.0092) were significantly larger than those of spring wheat (effect size = −0.45 ± 0.053; −0.34 ± 0.0095) (Q_B1_ = 13.53; Q_B2_ = 45.80). This indicates that compared with spring wheat, winter wheat had a more obvious effect of drought tolerance.

### Variations in GPC and GNC Response to Drought

**Figure 2:**
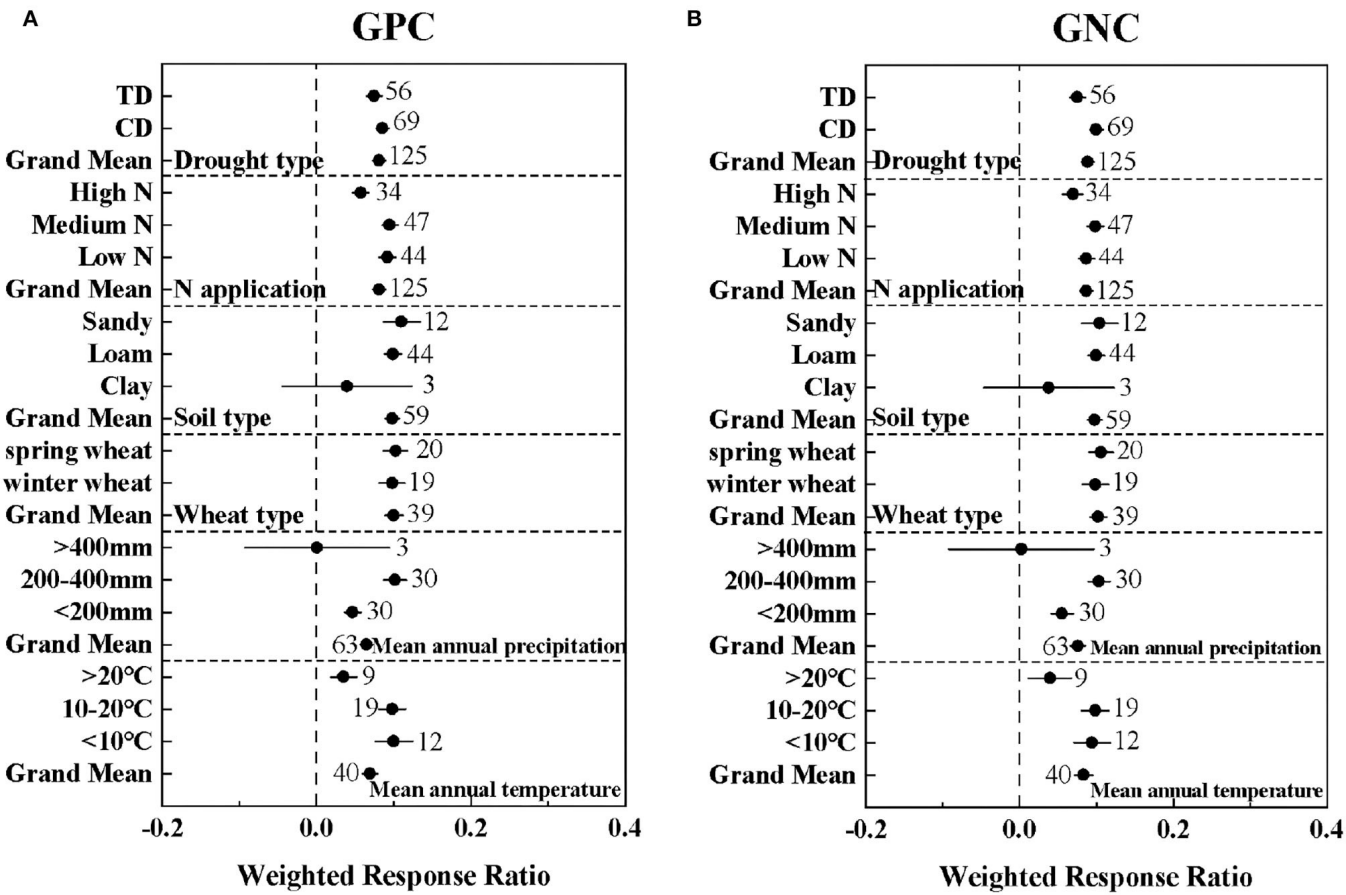
The effect of drought type (TD: Terminal drought stress; CD: Continuous drought stress), N application level [Low N (0–100 kg/ha); Medium N (100–200 kg/ha); High N (>200 kg/ha)], soil type, wheat type, mean annual precipitation, and mean annual temperature on the lnRRs of **(A)** GPC: grain protein concentration and **(B)** GNC: grain nitrogen concentration. The sample size of each variable is noted beside each bar. The effect of drought is significant if the ±95% confidence intervals of effect size do not overlap zero.

As shown in Figure 2, there was no significant difference in the effects of different wheat types and soil types on GPC and GNC (*P* > 0.05). In addition, both TD and CD significantly improved the GPC and GNC of wheat, but there was no significant difference between the different drought types in GPC. Nitrogen fertilizer significantly increased GPC, and the protein content reached a maximum at medium nitrogen levels (Figure 2A). The climate conditions of <10°C and 200–400 mm (effect size = 0.10 ± 0.024; 0.10 ± 0.014) had the greatest promotion effect for GPC and GNC (Figure 2). The positive drought response values of spring wheat to GPC and GNC (effect size = 0.10 ± 0.016; 0.11 ± 0.015) were greater than those of winter wheat (effect size = 0.0.98 ± 0.017; 0.098 ± 0.017), but the difference between the two was not significant (Q_B1_ = 0.18; Q_B2_ = 0.48).

### Controlling Factors of the Variations in GY and GPY Response to Drought Stress and Partial Correlation Coefficients (*r*)

**Figure 3:**
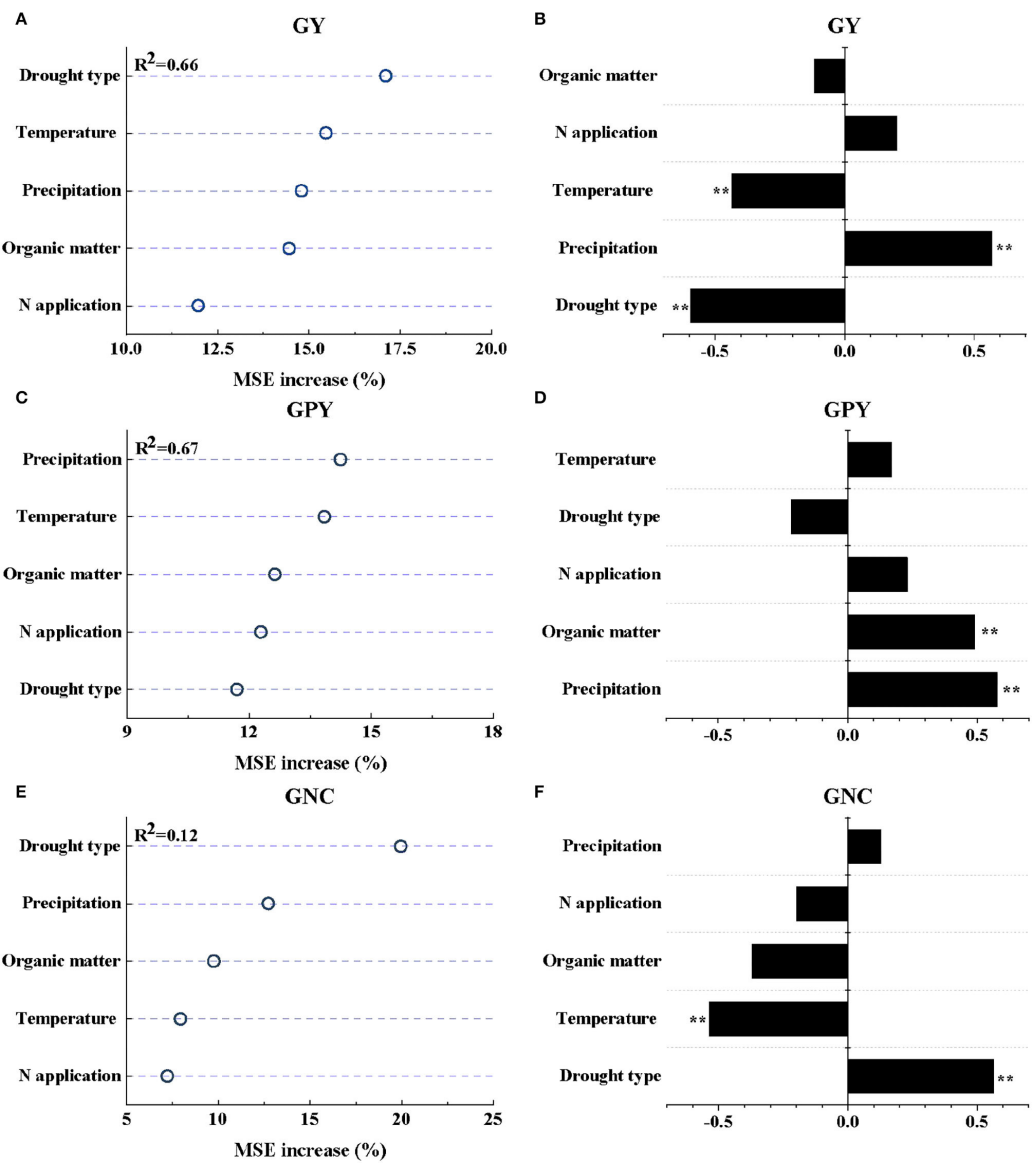
The relative importance of independent variables for the change of GY **(A)**, GPY **(C)**, and GNC **(E)** after drought stress as determined using random forests (RF) models. The partial coefficient (*r*) between each independent variable and GY **(B)**, GPY **(D)**, and GNC **(F)**. ** represents a significance at the 0.01 probability levels.

The results of the RF model analysis showed that 65.82 and 67.23% of the variation in the responses of GY and GPY to drought stress were explained by the factors of climates, soil organic matter, and N application (Supplementary Table S2; Figures 3A,C). It can be seen from the amplitude of increase in MSE that the change in GY response to drought was mainly explained by climatic factors (drought type + temperature + precipitation = 47.36%) (Supplementary Table S2). Among them, drought type had the largest contribution to GY response for drought (17.10%), followed by temperature (15.47%) and precipitation (14.79%) (Figure 3A). However, the contribution of the N application rate to the GY response to drought was the smallest (11.97%). In addition, drought type, precipitation, and temperature had extremely significant partial correlations with the drought response of GY (*P* ≤ 0.01), with partial correlation coefficients of −0.60, 0.57, and −0.44, respectively (Figure 3B).

The responses of GPY to drought stress were explained by the factors of climates, soil organic matter, and N application (Figures 3A,C). It can be seen from the amplitude of increase in MSE that the change in GPY response to drought was mainly explained by the factors of climate and soil factors (precipitation + temperature + soil organic matter content = 40.72%) (Supplementary Table S2), which explained 14.25, 13.84, and 12.63% of the change in GPY response to drought, respectively (Figure 3C). Except for drought type, all other factors were partially positively correlated with GPY. The annual mean precipitation and soil organic matter content were extremely significantly correlated with the response of GPY to drought (*P* ≤ 0.01), with partial correlation coefficients of 0.58 and 0.50, respectively (Figure 3D).

### Controlling Factors of the Variations in GPC and GNC Response to Drought Stress and Partial Correlation Coefficients (*r*)

The results of RF model analysis showed that the variation of GNC response to drought was mainly explained by climatic factors (drought type + precipitation = 32.68%) (Supplementary Table S2; Figure 3E). Among them, drought type had the largest contribution to the variation of GNC response (19.95%), followed by temperature (12.73%). However, the RF model explained only 1.24% of the variation in GPC responses to drought (Supplementary Table S2), so further studies were not carried out. GNC responses to drought type (*r*_1_ = 0.56) and temperature (*r*_2_ = −0.54) had extremely significant positive and negative partial correlations, respectively (Figure 3F; *P* ≤ 0.01).

### Effects of Drought on GY, GPY, and GNC

**Figure 4:**
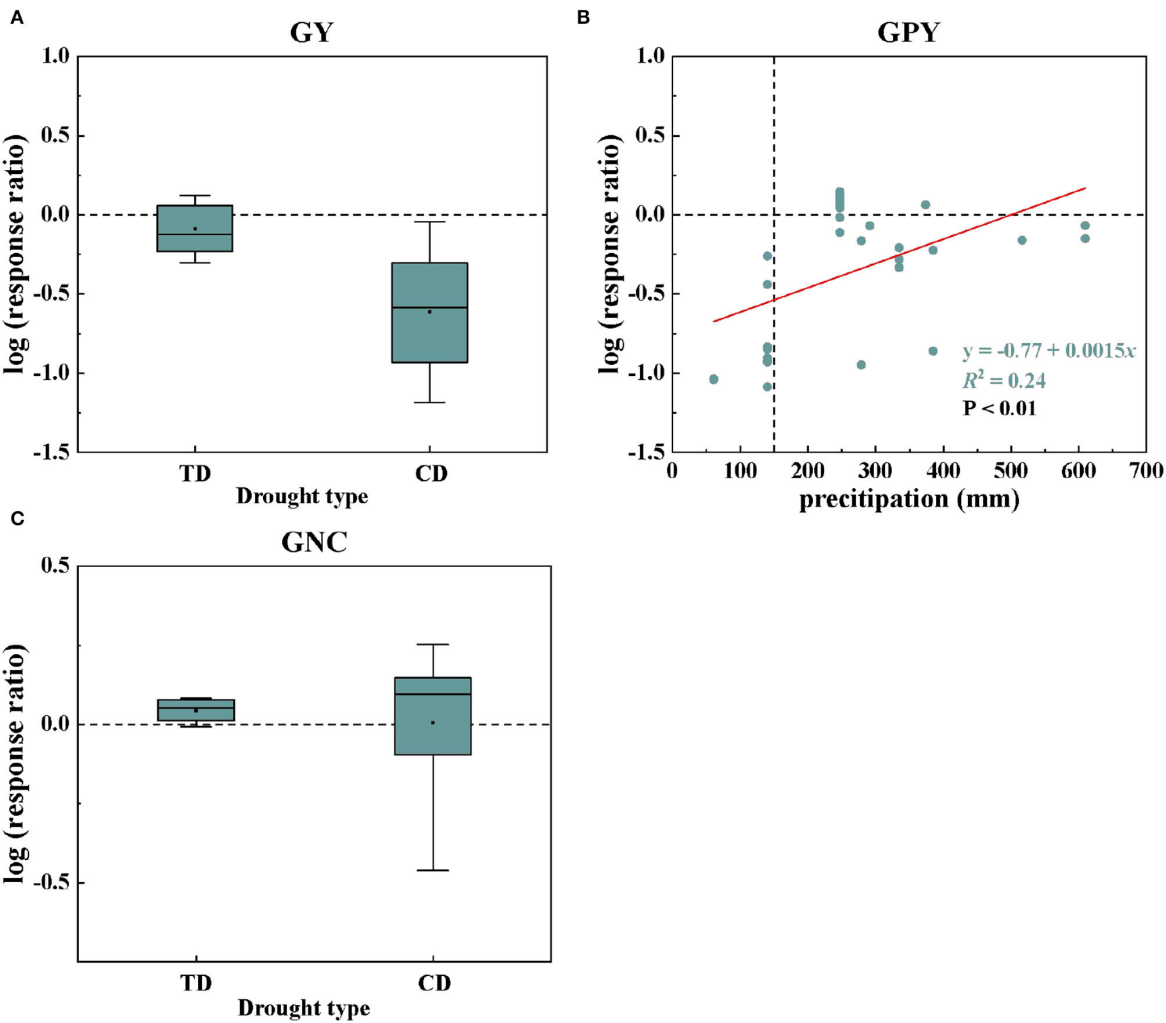
Changes in wheat GY **(A)** and GNC **(C)** responses to drought under different drought types; the relationships between the log conversed drought response ratios of GPY and precipitation **(B)**. In GPY, all data were fitted as a linear function.

From the abovementioned research results, it was found that the main factor controlling GY and GNC was of drought type, and the main factor controlling GPY was the precipitation (Figure 3). The log values of the GY and GNC responses to drought under different drought types are shown in Figures 4A,C. Compared with TD, CD significantly reduced the drought response value of GY, ranging from −0.089 to −0.61. However, CD significantly increased GNC drought response values ranging from 0.044 to 0.12. The relationships between the log conversed drought response ratios of GPY and precipitation clearly showed that it was linear (Figure 4B, *R*^2^ = 0.24). The drought response of GPY showed an upward trend with increasing precipitation, and the negative effect value of drought was completely eliminated when the precipitation reached 513.33 mm (Figure 4B).

## Discussion

This meta-analysis provided a comprehensive and quantitative analysis of the effects of drought on wheat yield, protein yield, grain protein content, and nitrogen content on a global scale, and identified the main environmental factors affecting the response to drought. Drought is an abiotic stress that severely limits global crop yields and challenges food security (Lesk et al., 2016).

The statistical model confirmed that wheat yield and protein content under drought conditions were affected by climatic factors (temperature, precipitation, and drought type), soil factors (soil type, soil organic matter content), and nitrogen fertilizer input. Among them, the responses of wheat GY and GNC to drought were mainly related to the drought type, while wheat GPY was mainly related to the precipitation (Figure 3). Some studies have explored the effect of different drought stages on crop yield and have shown that drought stress at the flowering stage leads to a more severe reduction in grain yield than in other periods, and long-term drought stress is more detrimental than single-stage stress (Nam et al., 2001; Yavuz et al., 2021). Meanwhile, another study has shown that the reduction in yield of drought stress in the reproductive growth stage is greater than that in the vegetative growth stage (Mi et al., 2018). In this meta-analysis, CD represented continuous drought stress, while TD represented terminal drought stress. The yield reduction due to CD (83.60%) was significantly greater than that of TD (26.43%) (Supplementary Table S3). The reason for this phenomenon may not only be due to the long-term drought stress of CD, but also mainly because CD contains the drought-sensitive flowering period, which reduces the total dry matter accumulation, and the long-term drought stress accelerates the shedding of plant parts (Mi et al., 2018; Ichsan et al., 2021). For grain protein and nitrogen content, drought significantly decreased GPY by 46.04%, but significantly increased GPC and GNC by 9.38 and 9.27%, respectively (Supplementary Table S3). Several studies have shown that water deficit is a major challenge to crop yield and quality under drought conditions. Insufficient water supply results in the reduction of carbohydrate synthesis of crops, further resulting in lower grain yield and protein yield (Angus and Herwaarden, 2001; Selim et al., 2019). Seleiman et al. (2021) argued that insufficient precipitation input is often the main driver of drought, which was consistent with our conclusions. With the changes of temperature and precipitation, the adaptation changes of GY and GPY were relatively consistent (Figure 1), which may be due to the decrease in the absorption and utilization of nitrogen and phosphorus under drought, resulting in GY and GPY generally reduced (Wang et al., 2018). The negative effects of drought were significantly alleviated with increased precipitation (Figure 1B). However, according to the fitted linear relationship, this negative effect of drought on GPY was only completely eliminated when the precipitation was >513 mm (Figure 4B).

In addition, the drought response of wheat was also related to soil type, soil organic matter content, temperature, and nitrogen fertilizer input. In terms of soil texture, sandy soils significantly alleviated the negative impact of drought to the greatest extent (Figure 1), which may be due to the fact that the available water content of the plant in the clay soil is less than that of the sandy loam under drought, and the rooting depth of the plant is limited (Cannell et al., 1984). Organic matter in the soil enhances the soil's ability to withstand drought and is the key to sustainable food production (Bot and Benites, 2005). Renwick et al. (2021) also believed that soil organic matter can enhance maize drought resistance. But in our study, except for protein yield, soil organic matter content was negatively correlated with GY, GPC, and GNC. In addition, the temperature was also one of the important factors affecting the drought effect. Basso and Ritchie (2014) believed that drought led to extremely high temperatures but is not the main reason for the decline in maize yields, which was consistent with our results. A study on quinoa showed that high nitrogen application confers quinoa a certain degree of drought tolerance under the stimulation of drought, which enhances the stimulation of nitrogen on production (Alandia et al., 2016). However, in our study, a high nitrogen application level did significantly alleviate the negative effects of drought but was not the main factor affecting the drought response of wheat GY, GPY, GPC, and GNC (Figure 3). The negative effect of drought on GY could be alleviated by increasing nitrogen application, but this negative effect could not be eliminated compared with the control at the same nitrogen application level.

In this study, the GPC response to drought was not significantly different between different drought types, which may be due to the fact that prolonged drought increases nitrogen uptake by plants and plants accumulate more proline when they suffer drought during the vegetative growth period, which is a compound with low molecular weight that maintains protein content, resulting in CD having more nitrogen and protein than TD (Maggio et al., 2002; Wang et al., 2018). However, further conversion of nitrogen to protein is hindered due to the greater damage to grain yield by CD (Xu and Zhou, 2006). In addition, we found that medium levels of nitrogen application rates were more beneficial to the accumulation of GPC and GNC in wheat under drought conditions, which may be due to the fact that nitrogen in protein mainly comes from nitrogen stored in plant tissues, and nitrogen use efficiency is relatively high under low and medium N levels (Martre et al., 2003). It was worth noting that soil organic matter content was also one of the important factors for the wheat response to drought. Fahad et al. (2021c) believed that sustainable soil and land management is conducive to crop growth, and soil organic matter content is conducive to sustainable development. On the one hand, one problem, however, one issue is that both soil and fertilizer contain nitrate and ammonium, and different nitrogen forms may be transformed in the soil. However, owing to the fact that it was not reported in the studies, we were unable to assess the effect of nitrogen form. On the other hand, some of the abnormal data in GPC and GNC research may largely depend on the drought tolerance of the wheat genes (Akagawa et al., 2007).

The response of wheat yield and protein content to drought may also be related to the amount of nitrogen, water, or phosphorus availability in soil (Rütting and Andresen, 2015; Kimball, 2016). However, the conclusions of the meta-analytic methods used in this study were limited by the database, which only included experiments from 15 different countries on 6 continents. Therefore, it was not fully represented in regions with fewer data, and there were certain limitations and deviations in certain environmental conditions and regions. A growing number of regions provide research results, and the representativeness of the data set will increase in the subsequent analysis. As the seriousness of global drought has been on an upward trend, it is highly necessary to increase soil moisture and nitrogen fertilizer inputs in dry areas to control crop yields. However, the strategy to improve GY through increased fertilization under drought conditions may lead to the loss of nitrogen pollutants in soil, water, and atmosphere, leading to negative environmental consequences (Ahmad et al., 2014). So, further research will have to be carried out in order to minimize the loss of drought to GY and GPY.

Our results had remarkable implications for global crop production and food security. Drought has always been a worldwide problem, severely limiting global crop production, and future global climate change will make drought conditions more serious (Hammad and Ali, 2014). Our findings had the potential to help researchers and decision-makers to make better decisions about crop production for different types of drought in different regions. In addition, cereal proteins include two categories of structural metabolic proteins (gliadins and glutenins) and storage proteins (albumin and globulins) (Zhou et al., 2018). The content and function of the four protein components are different. Therefore, in order to better reflect the quality of wheat protein, future research should use the method of meta-analysis to clarify the influence of external environmental factors on the content of four protein components and protein physicochemical properties of wheat under drought.

## Conclusion

Drought significantly decreased GY and GPY by 57.32 and 46.04%, but significantly increased GPC and GNC by 9.38 and 9.27%, respectively. The responses of wheat GY and GNC to drought were mainly related to the drought type, while the GPY was mainly related to the precipitation. The yield reduction due to CD (83.60%) was significantly greater than that of TD (26.43%). The relationship between the precipitation and GPY increased in accordance with linear functions, and this negative drought effect was completely eliminated when the precipitation was more than 513 mm. Sandy soils significantly mitigated the negative effects of drought to the greatest extent. High nitrogen application level significantly alleviated the negative effect of drought, but was not the main factor affecting the drought response of wheat. Compared with spring wheat, the drought resistance effect of winter wheat was more obvious.

## Data Availability Statement

The original contributions presented in the study are included in the article/Supplementary Material, further inquiries can be directed to the corresponding author/s.

## Author Contributions

CW: conceptualization, data curation, and writing—original draft. PD: conceptualization and software. LG: investigation. JW: investigation, methodology, and resources. JT: validation and visualization. XQ: validation and editing. BF: writing—review and editing. JG: conceptualization, supervision, funding acquisition, and writing—review and editing. All authors contributed to the article and approved the submitted version.

## Funding

This study was supported by the National Key R&D Program of China (2020YFD1000805), National Natural Science Foundation of China (31671631), Minor Grain Crops Research and Development System of Shaanxi Province (2016–2019), and Focus on Research and Development of Science and Technology Plan Projects in Shaanxi Province (2018 TSCXL-NY-03-04).

## Conflict of Interest

The authors declare that the research was conducted in the absence of any commercial or financial relationships that could be construed as a potential conflict of interest.

## Publisher's Note

All claims expressed in this article are solely those of the authors and do not necessarily represent those of their affiliated organizations, or those of the publisher, the editors and the reviewers. Any product that may be evaluated in this article, or claim that may be made by its manufacturer, is not guaranteed or endorsed by the publisher.
